# Identification of *Hymenolepis diminuta* Cysticercoid Larvae in *Tribolium castaneum* (Coleoptera: Tenebrionidae) Beetles from Iran

**Published:** 2017-05-27

**Authors:** Mahsa Sadat Makki, Gholamreza Mowlavi, Farideh Shahbazi, Mohammad Reza Abai, Faezeh Najafi, Bibi Razieh Hosseini-Farash, Salma Teimoori, Hamid Hasanpour, Saied Reza Naddaf

**Affiliations:** 1 Department of Medical Parasitology and Mycology, School of Public Health, Tehran University of Medical Sciences, Tehran, Iran; 2 Department of Medical Entomology, School of Public Health, Tehran University of Medical Sciences, Tehran, Iran; 3 Department of Parasitology and Mycology, Research Center for Skin Diseases and Cutaneous Leishmaniasis, School of Medicine, Mashhad University of Medical Sciences, Mashhad, Iran; 4 Centre of Excellence for Therapeutic Proteins and Antibody Engineering, Department of Parasitology, Faculty of Medicine, Siriraj Hospital, Bangkok, Thailand; 5 Department of Parasitology, Pasteur Institute of Iran, Tehran, Iran

**Keywords:** *Hymenolepis diminuta*, Cysticercoid, *Tribolium castaneum*, Iran

## Abstract

**Background:**

*Hymenolepis diminuta* is a cestod of rodents and rarely infects humans. Infection in humans is via ingestion of infected insects. This study was aimed to detect *H. diminuta* cysticercoids in red flour beetles, *Tribolium castaneum,* and cockroaches originated from different regions of Iran.

**Methods:**

The red flour beetles and cockroaches were collected from local bakeries in five cities including Tehran, Ahvaz, Kazerun, and Sabzevar during 2010–2011. Some beetles and cockroaches were colonized in insectary and adults from F1 generation were fed on *H. diminuta* eggs. Both laboratory-infected and field-collected samples were dissected and examined for cysticercoids. Detection of *H. diminuta* DNA in *T. castaneum* beetles was performed by targeting a partial sequence of Ribosomal gene.

**Results:**

Except the beetles from Ahvaz, all specimens were negative for cysticercoid by microscopy. Of the four dissected beetles from Ahvaz, one harbored 12 cysticercoids. Also, 110 (52%) of laboratory-infected beetles showed infection with an average of 12–14 larvae. None of the cockroaches was infected. Two beetles from Ahvaz, including the remainder of the microscopic positive specimen, yielded the expected amplicon in PCR assay. The *H. diminuta* DNA sequences generated in this study were identical and matched 97–100% with similar sequences from GenBank database.

**Conclusion:**

Lack of infection in the majority of beetles may reflect a low rat infestation rate in those areas, alternatively, the examined specimens might not have been the representative samples of the *T. castaneum* populations.

## Introduction

Human hymenolepiasis is caused by two cestodes, *Hymenolepis nana*, and *H. diminuta* (Wiwanitkit 2004, Magalhaes et al. 2013). There is a single report on mixed infection of the third species *H. microstoma* with *H. nana* in remote communities in the northwest of Western Australia (Macnish et al. 2003).

The tapeworm, *H. nana* is the primary cause of human infection, whereas *H. diminuta* rarely infect humans, and so far, only a few hundred cases have been reported (Tena et al. 1998, Wiwanitkit 2004). However, with increased awareness of the disease and improvement of laboratory techniques, more cases become apparent. Although the identified cases are mostly children, the disease can be seen in every age group (Cohen 1989, Tena et al. 1998, Marangi et al. 2003). Unlike, *H. nana*, transmission of *H. diminuta* requires an arthropod intermediate host to complete its life cycle. When *H. diminuta* fertilized eggs are excreted in the stool of an infected definitive host, they are ingested by various arthropods. Once inside the insect body, the oncospheres are released from the eggs, penetrate the intestinal wall of the host and develop into cysticercoid larvae. Soon after the ingestion of infected arthropod by the mammalian host, the cysticercoid larvae are released in the stomach, and small intestine and their life cycle is completed. The maturation occurs within 20–25d, and the adult worms can reach an average of 30cm in length. Humans are accidentally infected via ingesting the arthropods carrying cysticercoids.

Various arthropod species including flour beetles, moths, earwigs, and flea larvae may serve as the intermediate host. The red flour beetle, *Tribolium castaneum*, is an efficient intermediate host of this cestode (Bisseru 2013). It is a worldwide stored food pest and attacks grain products including flour, cereals, pasta, biscuits, beans, and nuts. The adults are long-lived and may live for more than three years.

This study was aimed to detect *H. diminuta* cysticercoids in *T. castaneum* beetles and cockroaches collected from local bakeries in five cities of Iran by microscopy and PCR assay. We also discuss the susceptibility of these beetles along cockroaches to *H. diminuta* infection under laboratory condition.

## Materials and Methods

### Collection of *Tribolium castaneum* beetles

Bakeries in different cities including Tehran in upper center, Ahvaz in the southwest, Iranshahr in the southeast, Kazerun in south, and Sabzevar in the northeast of the country were searched for red flour beetles during 2010–2011. The collected samples were kept in tubes containing flour at 28–30 °C and humidity of 20–40%. The collected insects were identified to the species based on morphological features using diagnostic keys (Bosquet 1990). Similarly, some cockroaches were collected from the same bakeries in Tehran.

### *Hymenolepis diminuta* eggs

*Rattus norvegicus* rats were captured from rat-infested areas in Tehran. The animals were euthanized, sacrificed, and mature worms were collected from the intestine. Posterior gravid proglottids of the worms were removed and dissected in normal saline to free the eggs.

### Laboratory-infected beetles

Flour beetles, *T. castaneum*, and American cockroaches, *Periplaneta americana*, were reared in the insectary at 28–30 °C with relative humidity of 20–40% and light and dark cycle of 12h/12h. The adult progenies from the F1 generations were allowed to feed on dough contaminated with *H. diminuta* eggs. After three weeks, the cockroaches and beetles were dissected in normal saline and examined for cysticercoids under a stereomicroscope with a magnification of 40X (Makki et al. 2011).

### Identification of cysticercoids in beetles by microscopy

We used 154 field-collected flour beetles (30 specimens from Tehran, 60 from Iranshahr, 30 from Kazeroun and Sabzevar each, and four from Ahvaz), 50 field-collected American cockroaches from Tehran, 210 laboratory-infected beetles, and 40 American and 70 German laboratory-infected cockroaches in this study. All the insects were dissected in normal saline and examined for cysticercoid larvae under a stereomicroscope with a magnification of 40X.

### PCR and sequencing

DNA extraction was performed on individual and pools of 20–50 beetles of laboratory-infected beetles as well as field-collected samples by phenol-chloroform method followed by ethanol precipitation as described by others (Ballinger-Crabtree et al. 1992). A partial ribosomal DNA sequence of *H. diminuta* (spanning the 3′ end of the 18S rRNA gene, internal transcribed spacer 1 (ITS1), 5.8S, ITS2 and the 5′end of the 28S rRNA gene) was targeted using the primers HF1 5′-gcggaaggatcattacacgttc-3′ and HR1 5′-gctcgactcttcatcgatccacg-3′ designed by others (Macnish et al. 2002). The 25μl reaction mixture contained 20pmol of each primer, 2.5mM MgCl_2_, 10mM Tris-HCl, 50mM KCl, 200 M of dNTPs, 1U of *Taq* polymerase, and 3μl of DNA. All amplifications were programmed for an initial denaturation step for 5 min at 94 °C, followed by 30 cycles of 94 °C for 30s, 63 °C for 40s, and 72 °C for 45s, with a final step of 10min at 72 °C. For field-collected samples, the annealing temperature was reduced to 61 °C and the cycles increased to 32. In all amplifications, DNA from *H. diminuta* and *H. nana* adult worms were included as positive controls and DNA of adult *Taenia* sp. as the negative control. The PCR products were run on 1% agarose gel, stained with ethidium bromide and visualized under UV. Amplicons from one field-collected beetle and *H. diminuta* and *H. nana* adult worms were sequenced in both directions using the same primers used for amplification and compared with similar sequences from GenBank database.

The data for sequences were submitted to GenBank database with accession numbers KJ917784 and KJ917785-7 for *H. nana* and *H. diminuta*, respectively.

## Results

### Microscopy

**Fig. 1.**
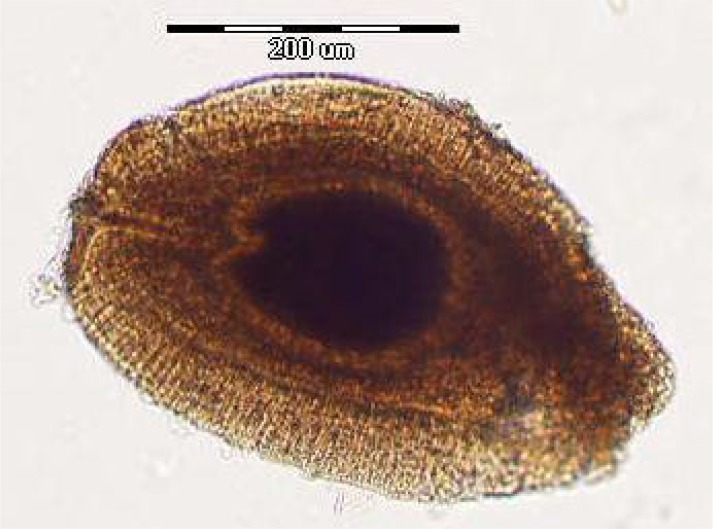
An *H. diminuta* cysticercoid derived from a *T. castaneum* beetle collected from Ahvaz, Iran.

Except for *T. castaneum* beetles from Ahvaz, all the specimens were negative for *H. diminuta* cysticercoids by microscopy. Out of four dissected beetles from Ahvaz, one harbored 12 *H. diminuta* cysticercoids (Fig. 1). Also, of the 210 *T. castaneum* beetles fed on food contaminated with the tapeworm eggs, 110 developed cysticercoids with an average of 12–14 larvae in insects. None of the laboratory-reared cockroaches fed in similar fashion turned positive.

### PCR and sequencing

**Fig. 2.**
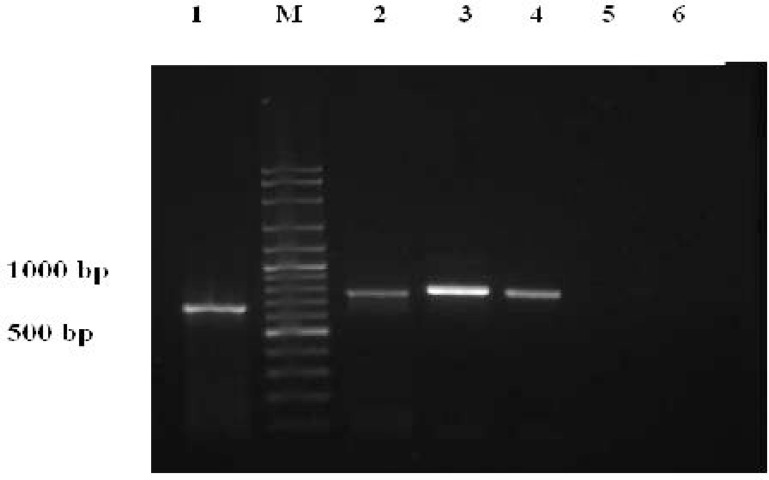
Amplification of a partial ribosomal DNA sequence (spanning the 3’ end of the 18S rRNA gene to the 5’end of the 28S rRNA gene) from *Hymenolepis* spp. 1, *H. nana* adult worm, M, 100bp marker, 2, *H. diminuta* adult worm, 3, infected beetle, 4, cysticercoid larvae, 5, *Taenia* sp, 6, negative control.

PCR amplification of rDNA gene from *H. nana* and *H. diminuta* adult worms yielded the expected bands of 645bp and 750bp, respectively. The 750bp band diagnostic for *H. diminuta* was observed in the pools of laboratory-infected *T. castaneum* beetles. Our PCR method could detect the DNAs equivalent to one and three cysticercoids in pools of 20 and 50 non-infected beetles, respectively. DNAs from 35 individual and ten pools of 20–50 beetles collected from different areas were negative for *H. diminuta*. However, DNA samples of two individual beetles from Ahvaz including the remainder of the microscopic positive specimen yielded the expected 750bp amplicon, implying the presence of *H. diminuta* in the insects (Fig. 2). The *H. diminuta* ribosomal DNA sequences generated in this study were identical and showed 97%–100% homology with three *H. diminuta* gene sequences from GenBank database (accession numbers, AF461125, JN258039, and JN258038) over 94%–100% of nucleotides.

## Discussion

The rat tapeworm, *H. diminuta*, is a cestode of rodents. In Iran, it is commonly found in rats and mice (Kia et al. 2001, Meshkekar et al. 2014, Yousefi et al. 2014). Recently, it was found in *Tatera indica* from southeast of the country (Nateghpour et al. 2015). This species may also affect pet animals like squirrels (*Callosciurus prevosti*) (d’Ovidio et al. 2015) and laboratory rats (Sreedevi et al. 2015). *Hymenolepis diminuta* infection in humans is very rare, but with increased awareness of the disease and the ability to identify different species of the genus *Hymenolepis*, more cases are becoming apparent. Infection of *H. diminuta* has recently been reported from various countries including Turkey, Tamil Nadu, Sri Lanka and India (Kalaivani et al. 2014, Sinhabahu et al. 2014, Tiwari et al. 2014, Kılınçel et al. 2015). In Iran, The last human infection was identified in a child in 2008 (Mowlavi et al. 2008). Infection with this worm is commonly light and self-limiting and deworming can be achieved with praziquantel as the drug of choice (Karuna and Khadanga 2013).

The flour beetles belonging to the genera *Tribolium* and *Tenebrio* are among the known intermediate hosts of this worm. They are adapted to survive in arid environments and are highly resistant to insecticides. The susceptibility to *H. diminuta* infection may vary among the members of these two genera, and with their life stage. In samples collected from a riding stable in Quebec, the intensity of cysticercoids in *Tenebrio obscurus* was higher than *T. molitor* and adults were more susceptible to infection than larvae (Rau 1979). Also, *T. castaneum* was more vulnerable to the infection than *T. confusum* (Yan and Norman 1995).

In the present study, all the *T. castaneum* beetles from different cities, except Ahvaz, were negative for *H. diminuta* cysticercoid by microscopy and PCR assay. Examination of limited specimens from Ahvaz revealed infection in two beetles. This finding suggests a high infection rate among the beetles, which can maintain the enzootic cycle. An earlier study in this city revealed high helminthic infection rates, particularly with Spirurids, among *R. norvegicus* rats. Out of 72 dissected rats, 8 (11.1%) were infected with *H. diminuta* (Kia et al. 2001). In the present study, 52% of the *T. castaneum* beetles fed on dough contaminated with *H. diminuta* eggs developed cysticercoids, this reflects their high susceptibility to hymenolepiasis, and their potency to serve as an efficient intermediate host. Unlike *T. castaneum* beetles, in none of the cockroaches fed in similar manner cysticercoids developed. Cockroaches have shown to be refractory to *H. diminuta* infection due to encapsulation of larvae by the insect hemocytes (Tu and Lai 2006). Prior infection of cockroaches with *Moniliformis moniliformis* may predispose them to *H. diminuta* infection. In cockroaches, a membranous envelope engulfs the acanthocephalan *M. moniliformis* and protects it from the hemocyte attack in the arthropod host (Holt 1989). The hatched oncospheres of *H. diminuta* may penetrate this envelope and, once inside, utilize its protective function to develop.

## Conclusion

Lack of infection in the majority of beetles may reflect a low rat infestation rate in those areas, alternatively, the examined specimens might not have been the representative samples of the *T. castaneum* populations. Further studies with inclusion of more samples and other potential intermediate arthropod hosts are required to elucidate the biology and life cycle of *H. diminuta* in urban rats.
